# Multiplexed single-mode wavelength-to-time mapping of multimode light

**DOI:** 10.1038/ncomms14080

**Published:** 2017-01-25

**Authors:** Harikumar K Chandrasekharan, Frauke Izdebski, Itandehui Gris-Sánchez, Nikola Krstajić, Richard Walker, Helen L. Bridle, Paul A. Dalgarno, William N. MacPherson, Robert K. Henderson, Tim A. Birks, Robert R. Thomson

**Affiliations:** 1 Scottish Universities Physics Alliance (SUPA), Institute of Photonics and Quantum Sciences, School of Engineering and Physical Sciences, Heriot-Watt University, Edinburgh EH14 4AS, UK; 2 Institute of Biological Chemistry, Biophysics and Bioengineering, School of Engineering and Physical Sciences, Heriot-Watt University, Edinburgh EH14 4AS, UK; 3 Department of Physics, University of Bath, Claverton Down, Bath BA2 7AY, UK; 4 Institute for Integrated Micro and Nano Systems, School of Engineering, University of Edinburgh, Edinburgh EH9 3FF, UK

## Abstract

When an optical pulse propagates along an optical fibre, different wavelengths travel at different group velocities. As a result, wavelength information is converted into arrival-time information, a process known as wavelength-to-time mapping. This phenomenon is most cleanly observed using a single-mode fibre transmission line, where spatial mode dispersion is not present, but the use of such fibres restricts possible applications. Here we demonstrate that photonic lanterns based on tapered single-mode multicore fibres provide an efficient way to couple multimode light to an array of single-photon avalanche detectors, each of which has its own time-to-digital converter for time-correlated single-photon counting. Exploiting this capability, we demonstrate the multiplexed single-mode wavelength-to-time mapping of multimode light using a multicore fibre photonic lantern with 121 single-mode cores, coupled to 121 detectors on a 32 × 32 detector array. This work paves the way to efficient multimode wavelength-to-time mapping systems with the spectral performance of single-mode systems.

## Abstract (web summary)

Photonic lanterns are made by merging several single-mode cores into one multimode core. Here, the authors show this type of structure can both perform wavelength-to-time mapping of multimode states of light and couple such light to an array of single-photon avalanche detectors.

In many photonic application areas, single-mode optical waveguides are preferred over multimode waveguides for the transmission of light. This is because single-mode systems do not suffer from either inter-modal dispersion or inter-modal coupling. These effects can be highly detrimental in areas such as conventional telecommunications (where they reduce the bandwidth-length product of a fibre-optic link) and fibre-optic imaging (where they scramble the information required to generate a clear image). Yet, there are also areas where multimode waveguides are required. For example, photon-starved applications such as astronomy^1^ almost solely use multimode fibres, since they enable the efficient collection and transmission of multimode signals to instruments for analysis. Multimode fibres have also recently attracted renewed attention for advanced telecommunications using space division multiplexing (SDM), which seeks to exploit different spatial modes in the same optical fibre to increase data capacity^2^. Such SDM systems can even exploit strong inter-modal coupling to mitigate differential group delay, spatial mode dispersion, mode-dependent loss, mode-dependent gain and nonlinear impairments^2^,^3^.

The benefits of multimode waveguides, such as higher collection efficiencies and increased tolerances to misalignment, can be combined with the transmission and processing benefits of single-mode optical waveguides by harnessing the efficient multimode-to-single-mode coupling capabilities offered by photonic lanterns (PLs)^4^,^5^,^6^,^7^,^8^,^9^. In its most general form, a PL is a gradual, and ideally adiabatic, transition between a multimode waveguide supporting *N* modes and an array of *N* single-mode waveguides. The multimode end of the PL facilitates the efficient collection of incoherent multimode states of light, while the single-mode cores enable the benefits of single-mode confinement and transmission to be harnessed. PLs were originally developed for future applications in ground-based astronomy. There it is desirable to utilize complex single-mode fibre-Bragg-gratings for removing unwanted atmospheric lines from the celestial light of interest (OH-line suppression)^10^,^11^, but it is also essential to efficiently collect the multimode light that forms the telescope point spread function^12^. The applications of PLs are now moving well beyond astronomy. For example, PLs are attracting considerable interest in SDM telecommunications, where they enable the efficient coupling of light between multiple single-mode fibres and a few-mode fibre^13^,^14^,^15^. They are also of interest for applications such as coherent free-space communications and coherent light detection and ranging (LIDAR)^16^,^17^, where they facilitate greatly improved multimode signal collection along with excellent mode-matching to a single-mode local oscillator for clean heterodyne mixing.

Wavelength-to-time-mapping (WTM) is a phenomenon that occurs when a wave packet of light (or indeed any other type of wave) propagates through a sufficiently long length of a dispersive medium. As shown conceptually in Fig. 1a, WTM occurs in fibre optics when a short pulse of broadband light propagates along a length of dispersive optical fibre. The input pulse can be considered as being composed of an infinite set of pulses with different central wavelengths, each of which propagates at a different group velocity. As discussed in ref. 18, after propagating down a length of fibre, two such pulses with different central wavelengths (*λ*_1_ and *λ*_2_) will become separated by a temporal duration (Δ*t*), according to equation (1):


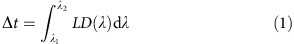


where *L* is the length of the single-mode fibre and *D* is the group velocity dispersion (GVD) of the fibre. Thus wavelength information is converted into arrival-time information.

WTM is an established technique in optics, where it has already found applications in real-time spectroscopy^18^,^19^,^20^,^21^. For example, WTM has provided significant insights into the nonlinear dynamics and wavelength correlations that occur during broadband supercontinuum generation, insights that would simply not be possible using measurements that are averaged over multiple pulses^22^. WTM is now also beginning to find real-world applications in areas such as Raman spectroscopy where, in combination with time-correlated single-photon counting (TCSPC), it enables the acquisition of Raman spectra without the requirement for a conventional spectrometer^18^,^23^,^24^. To date, however, all work on WTM-based TCSPC Raman spectroscopy has been limited to the use of one single-photon detector, restricting the signal acquisition rates possible. One option to address this is to use a single-photon avalanche detector (SPAD) array for multiplexed detection. The Megaframe (MF32) is a particular example of such an array, which has recently attracted considerable attention for applications in areas such as fluorescence lifetime^25^ and light-in-flight^26^ imaging. Each SPAD on the Megaframe has its own time-to-digital converter (TDC) for TCSPC^27^,^28^,^29^. To achieve acceptable dark-count rates, the photosensitive area of each SPAD on the Megaframe is only ≈6 μm in diameter. This, combined with the fact that each SPAD has its own dedicated TDC electronics, means that the SPADs are spaced on a 50 × 50 μm grid, and the SPAD array exhibits a physical fill factor of ≈1%.

In the present work, we demonstrate that multicore fibre (MCF)-based PLs can be used to efficiently and adiabatically couple multimode states of light to the Megaframe, and that the use of a PL for coupling light to the SPAD array can increase the effective fill factor of a subset of SPADs on the array from ≈1% to at least 46%, while also combining the benefits of multimode light collection and single-mode transportation and delivery. To showcase the enabling nature of the techniques we present here, we use a PL for multiplexed single-mode TCSPC-based WTM of multimode states of light. The application of MCF PLs to coupling multimode light to SPAD arrays may also prove to be enabling in many single-photon application areas. For example, with specific reference to the coherent free-space communication and coherent LIDAR applications mentioned above, the use of MCF lanterns and multi-pixel SPAD arrays offers a route to high multiplex gains, with a consequent increase in sensitivity and measurement speed. More generally, the splitting and reformatting capability offered by PLs could significantly increase data acquisition rates in single-photon counting applications that suffer from pulse pile-up^30^, by efficiently coupling the input signal to many SPADs in a scalable manner. In more fundamental areas of physics, the use of PLs for adiabatically coupling multimode states of light to multi-pixel SPAD arrays may also prove to be powerful in quantum optics, for observing multi-photon, multimode interference in high-dimensional quantum systems^31^.

## Results

### Experimental set-up

**Figure 1:**
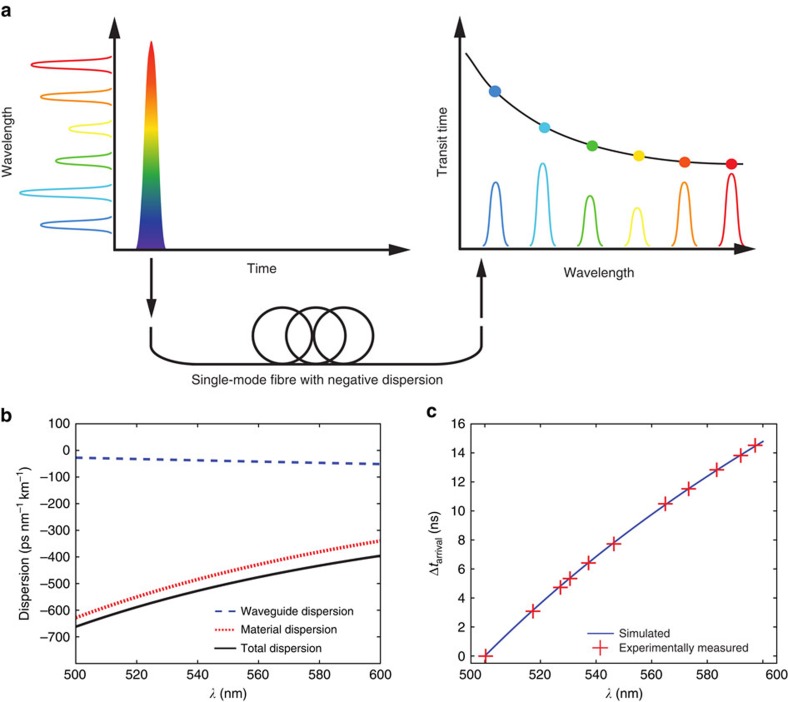
Wavelength-to-time mapping in single-mode optical fibre. (**a**) Conceptual representation of the wavelength-to-time mapping phenomenon in fibre optics. On the left of the figure, a short broadband pulse is coupled into a long length of single-mode fibre. On the right of the figure, the different colours that form the input pulse emerge from the fibre at different times due to the chromatic dispersion of the fibre, resulting in the mapping of wavelength to arrival time. (**b**) Simulated dispersion of the MCF cores. Also shown are the approximate contributions due to waveguide and material dispersion. (**c**) Expected differences in arrival times Δ*t*_arrival_ after propagating along 290 m of the MCF, according to the dispersion profile shown in **b**. The crosses represent real data measured for the central core of the MCF. Note that larger values of Δ*t*_arrival_ indicate a shorter propagation time along the fibre.

**Figure 2:**
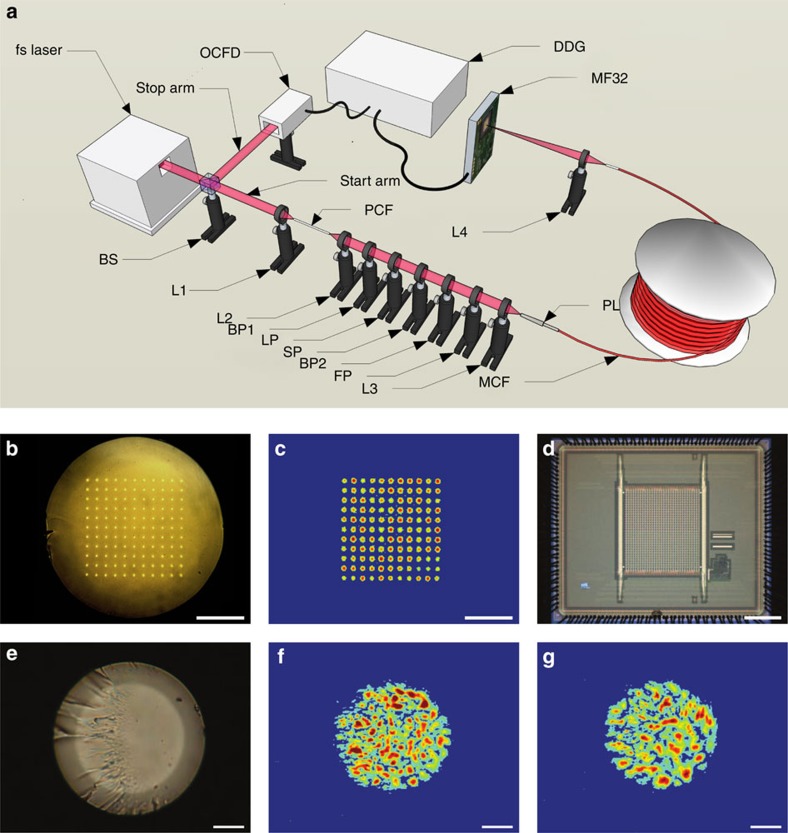
Experimental set-up and key components of the multiplexed single-mode wavelength-to-time-mapping system. (**a**) Experimental set-up used for multiplexed single-mode wavelength-to-time mapping. (**b**) Micrograph of the MCF, showing the 11 × 11 square array of single-mode cores. Scale bar (also in **c**), 50 μm. (**c**) False colour image of the output of a 9 m length of MCF when coupling 532 nm light into a PL at the opposite end. (**d**) Micrograph of the Megaframe detector (MF32) with 32 × 32 SPADs. Scale bar, 800 μm. (**e**) Micrograph of the multimode end of a PL. Scale bar (also in **f**,**g**), 10 μm. (**f**,**g**) False colour images of different output intensity profiles from **e** when exciting different single-mode MCF cores at the opposite end of the PL using 532 nm light.

The experimental set-up for the WTM experiments, and the key components used in this set-up, are presented in Fig. 2. For multiplexed single-mode WTM, we designed and fabricated a custom MCF. The MCF consists of an 11 × 11 approximately square array of single-mode cores, as shown in Fig. 2b. As detailed in ‘Methods' section, the MCF core positions are mapped optimally onto a square grid spacing of 10.53 μm, while the root-mean-square (RMS) displacement between the MCF cores and the grid points was found to be at most 0.54 μm. The MCF cores have a diameter of 1.63 μm, and are formed from germanium-doped silica. The cladding material is pure silica, the numerical aperture of the cores is 0.22 and the fibre's outer diameter is 200 μm. This design facilitates single-mode operation at wavelengths longer than *λ*=470 nm, with negligible core-to-core coupling for *λ*<610 nm after 300 m of propagation (see ‘Methods' section). Using near-field imaging, the MCF cores were observed to support a single mode at 550 nm, with a mode field diameter of ≈1.84 μm (see ‘Methods' section). This is in close agreement with the 1.93 μm value expected according to theory^32^. Figure 1b presents the simulated GVD of the MCF cores throughout the 500–600 nm region, where it can be seen that the total simulated GVD varies between −661.8 and −395.6 ps nm^−1^ km^−1^, respectively. The GVD simulations were performed using the refractive index dispersion data for undoped and Ge-doped silica as detailed in ref. 33 (samples 3 and 9, respectively, in Table 1 in ref. 33). On the basis of the simulated GVD, Fig. 1c presents the predicted difference in arrival times for pulses with different central wavelengths after propagating along a 290 m length of the MCF.

As shown in Fig. 2a, the experimental set-up for multiplexed WTM consists of two different arms for the TCSPC measurement. In the start arm, 1,064 nm femtosecond laser pulses at a repetition rate of 500 kHz are focused into a 20 cm long photonic crystal fibre^34^ using lens L1 to generate a broadband supercontinuum. The supercontinuum is collimated using lens L2 and projected through a series of spectral filters. First, the pump beam is blocked and the supercontinuum narrowed using a bandpass filter (BP1) that passes only light within the 400–700 nm range. For all measurements, the wavelength range of the supercontinuum was further limited to between *λ*_min_=500 nm and *λ*_max_=600 nm using the long-pass (LP) and short-pass (SP) filters. For calibration purposes, an angle-tuned Fabry-Pérot (FP) interference filter and bandpass filter (BP2) are also inserted into the beam path to produce spectrally narrow tuneable pulses of light. The filtered light is then focused using lens L3 into the multimode end of the PL^4^ (Fig. 2e), where it excites a coherent multimode state (Fig. 2f,g). The fabrication and loss characterization of the PL is detailed in ‘Methods' section. The PL transition then adiabatically couples this state to the 121 single-mode cores of a 290 m long length of MCF. As discussed in detail below, the 290 m length of fibre was chosen to achieve a WTM spectrum with a spectral resolution of ≈1 nm. At the other end of the MCF, the single-mode cores are directly imaged onto the Megaframe SPAD array (Fig. 2d) using lens L4, with the magnification and alignment carefully controlled to couple each MCF core to a separate SPAD. As an example, Fig. 2c presents the recorded output from a 9 m long MCF when light is coupled into the PL at the opposite end. The PL effectively increases the fill factor of a subset of SPADs on the Megaframe, by spatially rearranging the input light into a pattern, which can then be efficiently coupled to the photosensitive areas of each pixel. As detailed in the ‘Methods' section, we estimate the potential effective fill factor to be at least 46%, a significant increase on the ≈1% physical fill factor of the Megaframe itself. For the stop arm of the set-up, a small fraction of the fs-laser light is tapped using a beam splitter (BS) and coupled onto an optical constant fraction discriminator (OCFD). The OCFD generates a stable electrical stop pulse for the TCSPC. Also see ‘Methods' section for further details.

The Megaframe is a complementary metal oxide semiconductor (CMOS) SPAD array with TCSPC capability, consisting of a square array of 32 × 32 pixels, each with a ≈6 μm diameter photosensitive area and a pixel pitch of 50 μm. The photon detection efficiency of the SPADs is a function of wavelength, peaking at ≈500 nm and ranging between 18 and 28% depending on the excess bias voltage applied^35^. For our experiments, the excess bias voltage was set to 1.0 V, and a peak photon detection efficiency of 19% is expected, with a photon detection efficiency of >10% from ≈400 to 700 nm. The spectral dependence of the SPAD performance, together with the high GVD of silica in this region, guided our choice to perform the multiplexed WTM experiments in the 500–600 nm spectral region. As already stated, each pixel on the Megaframe has its own TDC, enabling the array to generate a photon arrival timestamp with a time resolution of 53 ps. Each timestamp is 10 bits long, resulting in a dynamic range of 54 ns. The Megaframe is designed to operate in reversed start–stop TCSPC mode. Here the detection of a single photon at the Megaframe starts the TCSPC measurement, and the trigger pulse generated by the OCFD stops the measurement. An electronic time delay using a digital delay generator is used to match the propagation time of the 290 m long MCF in the start arm. For the current architecture, each pixel can deliver 500,000 timestamps per second to the field programmable gate array^29^. Before conducting any WTM experiments, the Megaframe was characterized in terms of high dark count rate (DCR) pixels. It was found that ≈15% of the pixels possess a high DCR, and that these pixels are randomly scattered across the array. These pixels were removed from any post-processing of the WTM data. The instrument response function (IRF) of the instrument defines the maximum achievable timing resolution. Across the Megaframe, the full-width-half-maximum (FWHM) of the IRF varied from 137 to 174 ps, a variation that originates from the fact that each pixel has its own TDC. Another important characteristic of the Megaframe is how uniformly the IRFs for the SPADs align in time due to variations in the time delay of the stop signal. The standard deviation (s.d) of the variation in timing response about the mean was measured to be ±265 ps. This shift was taken into account during per-pixel calibration measurements.

### Calibration of the wavelength-to-time-mapping process

**Figure 3:**
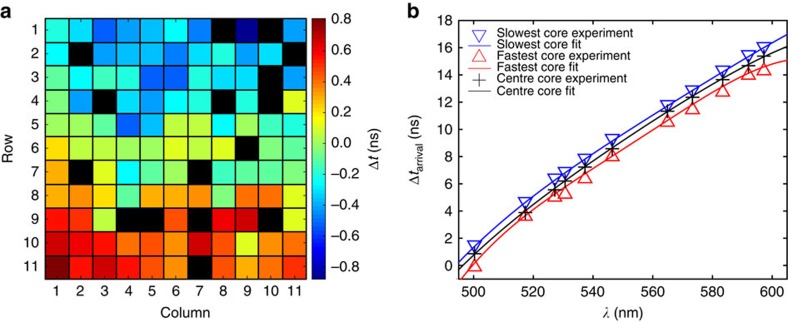
Calibration of arrival times for each fibre core. (**a**) Difference in arrival times (Δ*t*) for each core of the 290 m length of MCF measured at wavelength *λ*=531 nm. A black square indicates a pixel with a high DCR. (**b**) Measured (symbols) and fitted (solid lines) wavelength-dependent arrival times for the MCF cores with the highest and lowest group velocities, as well as the central MCF core.

To calibrate the WTM properties of the MCF, spectrally narrow pulses of light were selected from the supercontinuum. This was achieved by inserting the bandpass (BP2) and FP filters into the beam path. The FP could be angle-tuned to control the transmission wavelength, and the bandpass of BP2 was chosen such that only the light within the central peak of the FP was selected. Before each TCSPC measurement, a spectrum of the filtered light was obtained using a commercial spectrometer placed directly after the FP. Initially, light at *λ*=531 nm was coupled into the multimode end of the PL, which was attached to 290 m of MCF. The average propagation time along the MCF was measured to be ≈1.3 μs, while Fig. 3a presents the difference in arrival times (Δ*t*) recorded for the 11 × 11 array of MCF cores (black squares represent pixels with a high DCR). The maximum difference in arrival time was measured to be ≈1.7 ns, and it is clear that the group velocity varies across the fibre, exhibiting a maximum in the bottom left of the array and decreasing progressively across the MCF to the opposite corner. The precise reason for this variation is currently not known, but such differences could arise during the MCF fabrication, or are more likely due to spooling-induced strain across the MCF. These results immediately indicate that optimal multiplexed WTM using an MCF requires independent detection and calibration for each core of the MCF.

The WTM mapping process was then calibrated for each MCF core by angle tuning the FP to generate narrow band pulses across the 500–600 nm range. Figure 3b presents the WTM calibration curves across this range for the MCF cores with the highest and lowest group velocities, and also the central MCF core—the core that should be least affected by spooling-induced strain. Also shown in Fig. 3b are fourth order polynomial fits to the data. The equations of these fits are used in the following section to convert arrival time into wavelength. The arrival times for the central core are also plotted in Fig. 1c, where it can be seen that the WTM process in the central core is functioning in close agreement with the simulated dispersion profile of the MCF reported in Fig. 1b.

To probe the spectral resolution we can expect from our WTM system, we tuned the FP to generate narrow band pulses at *λ*=550.3 nm. When measured using a spectrometer with a FWHM spectral resolution of 0.1 nm, the FWHM passband of the filter at this wavelength was measured to be 0.5 nm, while the WTM data obtained from the central MCF core indicated that the pulses exhibited a FWHM spectral width of 1.2 nm. The spectral resolution of the WTM spectra obtained from the central core was then estimated by convolving the filter passband spectrum with a Gaussian profile of increasing width, until the FWHM of the convolved data matched the 1.2 nm FWHM of the WTM spectrum. This occurred when the Gaussian FWHM was set to 0.96 nm, and this represents the approximate equivalent linewidth of the WTM spectra obtained from the central MCF core at 550 nm. The spectral resolution of any WTM spectra is, according to equation (1), determined by the dispersion and length of the fibre, but also the IRF, which includes all instrument properties (the detector array, electronics and the laser source jitter) that degrade the temporal precision of the measurement. We can calculate the expected resolution of a WTM spectrum at some wavelength by considering how widely spaced two pulses must be in wavelength if they are to be separated by the FWHM of the IRF (≈150 ps). On the basis of the arrival times for the central MCF core presented in Fig. 3b, the dispersion is calculated to be ≈−505.9 ps nm^−1^ km^−1^ at 550.3 nm, and equation (1) indicates that the two pulses must be separated by 1.02 nm if they are to arrive separated by 150 ps. This value is close to the 0.96 nm resolution we evaluated experimentally using the FP filter, and further confirms our WTM instrument is operating as would be expected according to theory. As shown in Fig. 1b, the dispersion of the MCF cores is expected to increase (decrease) as we move to shorter (longer) wavelengths from 550 nm, and the central core is measured to exhibit a dispersion of −621.6 and −417.8 ps nm^−1^ km^−1^ at 510 and 590 nm, respectively. We would therefore reasonably expect the resolution of the WTM spectra obtained from this core to range from ≈0.8 to 1.2 nm according to equation (1). In future applications, the final spectrum from the multiplexed WTM system will be obtained by summing the spectra obtained from each MCF core. In this case, the line function of the final instrument at some wavelength would be defined by the addition of the linefunctions exhibited by each core.

### Broadband multiplexed wavelength-to-time mapping

**Figure 4:**
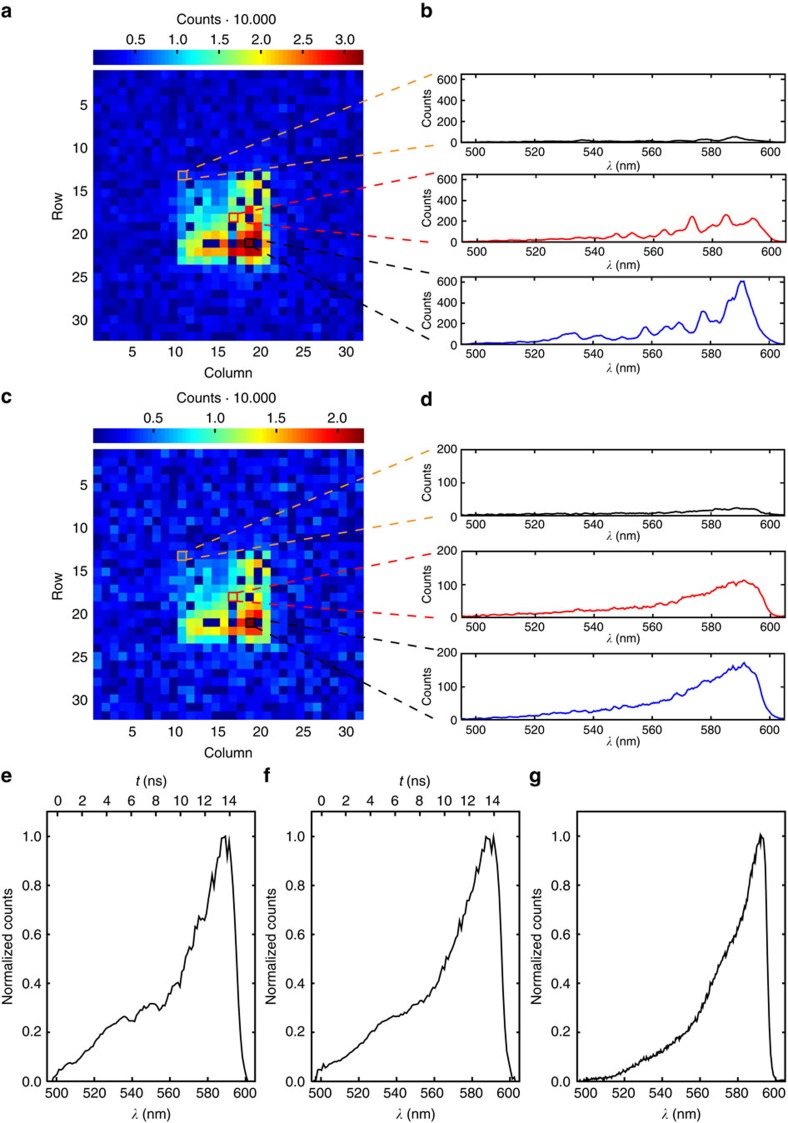
Recorded broadband wavelength-to-time mapped spectra. (**a**) Summed counts in each pixel of the Megaframe for the measurement without the rotating diffuser plate. (**b**) Wavelength-to-time mapped spectra for three representative pixels. The spectra are different due to wavelength-dependent coupling of the multimode light to the single-mode cores. (**c**) Summed counts in each pixel for the measurement with the rotating diffuser plate. (**d**) Wavelength-to-time mapped spectra for the same pixels selected in **b** are shown, but this time all spectra are comparable due to the equalized excitation of all modes in the PL over the duration of the measurement. Summed spectra obtained from all illuminated pixels for the measurement without the rotating diffuser plate (**e**), with the rotating diffuser plate (**f**) and a reference spectrum recorded with a conventional spectrometer (**g**). For the WTM spectra, the upper *x* axis shows the difference in arrival times across the spectra.

For broadband WTM measurements, the FP and bandpass (BP2) filters were removed, and the supercontinuum was spectrally narrowed to within the 500–600 nm range using only the BP1, SP and LP filters shown in Fig. 2a. Broadband WTM spectra were then obtained in two ways. For the first set of measurements, the supercontinuum was focused directly into the multimode end of the PL, and Fig. 4a presents a colour map of the total counts for each pixel across the Megaframe, together with WTM spectra for three representative pixels, as shown in Fig. 4b. When injecting the light into the PL in this manner, it was observed that each core at the end of the 290 m long MCF emitted light with very different spectra—the result of wavelength-dependent coupling in the multimode end of the PL. It is also apparent in Fig. 4a that the total counts across the 11 × 11 array varied significantly, and subsequent investigations (see ‘Methods' section) revealed that this observation was primarily the result of variations in the propagation losses of the MCF cores.

The fact that the distribution of light across the MCF cores at the output of the fibre is strongly wavelength dependent immediately presents an interesting issue for the final objective of performing multiplexed WTM on arbitrary multimode states. Ideally, all MCF cores would exhibit the same propagation loss and the same coupling efficiency to the SPAD array, and all SPADs would exhibit the same detection efficiency. But if this ideal situation is not achieved, there will be a wavelength-dependent loss of information in the final spectrum. Generally speaking, the importance of this will reduce as the number of modes in the measurement increases, since the fractional contribution to the final spectrum from any particular mode will reduce. To investigate how this loss of information has impacted the WTM spectra we measured, we performed a second set of WTM experiments, during which a rotating diffuser plate was placed directly in front of the multimode end of the PL. The effect of this diffuser plate was to excite all of the modes in the multimode end of the PL equally in a time-averaged manner, with the aim of exciting each single mode in the MCF equally at all wavelengths. In this case, all MCF cores should produce the same WTM spectrum—the same spectrum we should measure without the diffuser plate if there is no loss of information. Figure 4c presents a colour map of the summed counts obtained for each pixel across the Megaframe, while Fig. 4d confirms that under these illumination conditions, all MCF cores produce very similar WTM spectra.

Figure 4e,f, presents the final normalized WTM spectra for the measurements taken without and with the rotating diffuser plate, obtained by adding together the individual WTM spectra obtained from each MCF core, together with a spectrum of the MCF output obtained using a conventional spectrometer (Fig. 4g). As can be seen, the spectrum obtained without the diffuser plate is close to that obtained with the diffuser plate, but there are some additional features in the spectrum obtained without the diffuser plate due to loss of some spectral information. Both WTM spectra are also in general agreement with the spectrum measured using a conventional spectrometer, although there is a clear increase in signal at shorter wavelengths in the WTM spectra compared with the conventional spectrum. The precise reason for this is yet to be confirmed, but could be the result of a wavelength-dependent mode field diameter in the MCF, with shorter wavelengths being more confined to the core than longer wavelengths. In this case, since the SPADs are overfilled in our experiment, shorter wavelengths would be more efficiently coupled to the SPAD array than longer wavelengths. We also note that the photon detection efficiency of the Megaframe SPADs varies by ≈±15% of the mean across the 500–600 nm range^35^. Such variations would further contribute to the differences between the WTM spectra and the spectrum obtained with a conventional spectrometer, and would have to be taken into account in future applications.

## Discussion

We have demonstrated a new route to enable the efficient and adiabatic coupling of multimode states of light to multi-pixel SPAD arrays using MCF-based PLs. This approach is capable of increasing the effective fill factor for a subset of SPADs within the SPAD array from ≈1% to at least 46%, while also combining the benefits of multimode collection and single-mode transportation and delivery. In the future, the effective fill factor can be further increased via optimized MCF fabrication, to ensure the MCF cores lie precisely on a square grid, and we have shown in ‘Methods' section that doing so with the current MCF parameters could result in an effective coupling efficiency of 64%. To increase the effective fill factor even further, it would be necessary to further increase the overlap of the imaged MCF modes with the active area of the SPADs. To achieve this, one could increase the SPAD diameter, but this would come at the expense of increased dark counts. A more sensible approach would be to decrease the size of the guided mode itself in the MCF, by using smaller and higher refractive index contrast MCF cores.

Using our PL-based SPAD array coupling capability, we also demonstrated the multiplexed single-mode wavelength-to-time mapping of multimode states of light. We showed that wavelength-to-time mapped spectra measured without the rotating diffuser plate are broadly in line with that recorded using a commercial spectrometer, but that they also exhibit some artefacts due to high DCR pixels on the Megaframe and variations in the propagation losses of the MCF cores. We also demonstrated that these artefacts can be removed by using a rotating diffuser plate to equally excite the modes in the PL in a time-averaged manner. For future applications, it may be possible to mimic the scrambling function of the rotating diffuser plate in a more efficient manner, by integrating multiple PL transitions into the MCF and gently agitating the fibre. Such an approach has already been demonstrated to result in excellent mode scrambling^7^. We envisage that following the development of optimized MCF's and SPAD arrays, the techniques we have proposed and demonstrated here will open up new applications in photon-starved applications, such as Raman spectroscopy, coherent LIDAR, coherent free-space optical communications and quantum optics.

## Methods

### MCF core positions

To precisely measure the spatial arrangement of the MCF cores, one end of a length of MCF was flooded with white light, while the other end of the MCF was attached to a set of nm-precision x-y-z Aerotech stages. The output of the MCF was then imaged onto a camera with a high magnification, such that the relative positions of the MCF modes could be mapped by precisely translating the MCF end while monitoring the positions of the core modes on the camera.

After obtaining the relative MCF core positions, we then used computer simulations to quantify how much the MCF core positions deviate from a perfect square grid. An ideal 11 × 11 square grid of points was fitted to the measured MCF core positions by adjusting the rotation and dilation of the grid while keeping the central grid point on the central MCF core position. These parameters were optimized to minimize the RMS displacement between the MCF cores and the grid. This was achieved for a grid spacing of 10.53 μm, with an RMS displacement of 0.54 μm. This is an upper bound, since we did not attempt to optimize with respect to the translation of the central grid point.

### MCF core-to-core cross coupling

It was of particular importance to design a fibre that exhibited negligible cross coupling between adjacent cores after propagating along a 300 m length, as cross coupling would compromise any temporal information obtained in the WTM experiment. To verify that cross coupling was negligible, light at different wavelengths was coupled into a single core of a 300 m length of the MCF and the distribution of light at the output of the fibre was measured with a CCD camera (Thorlabs DCC1645C). No significant cross coupling for wavelengths below *λ*=610 nm was observed.

### Mode-field diameters of the MCF cores

The modes of the MCF cores were characterized at 550 nm using calibrated near-field imaging. It was found that the MCF cores exhibited a 1/*e*^2^ mode field diameter of 1.83±0.04 μm in one axis, and 1.85±0.05 μm in the other, where the axis are aligned with the axes of the MCF square core array and the quoted uncertainties are the s.d.'s of the measured distributions of mode sizes. These values are in close agreement with the theoretical value of 1.93 μm expected from the MCF design^32^.

### MCF propagation loss

**Figure 5:**
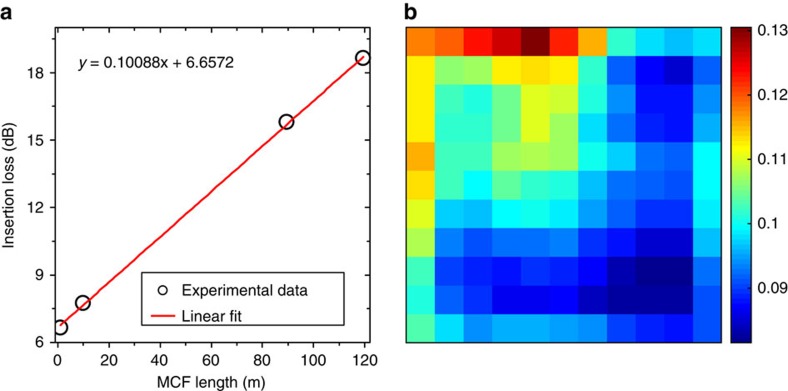
Propagation losses of the MCF cores. (**a**) Insertion loss of the central MCF core as a function of MCF length. The red line is a linear fit to the experimental data points. The equation of the fit is also given. (**b**) Spatial map of the attenuations across the 11 × 11 array of MCF cores. The units of the colour map are dB m^−1^.

The cutback technique was used to determine the propagation losses of each MCF core. Light at 550 nm (selected from an NKT Photonics SuperK Extreme EXW-12 supercontinuum source) from a single-mode fibre was imaged onto the facet of one end of 119.5 m of the MCF, with a sufficiently high numerical aperture to ensure that only one core was excited at a time. The input coupling end of the MCF was mounted on computer controlled x-y-z translation stages with ≈nm resolution, ensuring that any core of the MCF could be repeatably excited with high precision. After misaligning the input coupling, optimal coupling could be re-achieved with a ±0.5% variation in the output power from MCF at the opposite end, indicating that variations in input coupling during the cutback measurements have a negligible impact. The power of light emerging from the output end of the MCF was then measured when coupling light into each of the 121 MCF cores individually. This process was then repeated for MCF lengths of 89.5, 10 and 0.97 m. In all cases, the MCF was removed from the output end while keeping the input end mounted on the high precision stages. As an example, Fig. 5a presents the results of the cutback measurements for the central core of the MCF, which exhibits an attenuation of 0.1 dB m^−1^. The linear fit to the data in Fig. 5a is extremely good, with an *r*^2^ (coefficient of determination) of 0.99954 and a residual sum of squares of 0.0324, dB^2^ across the four experimentally evaluated points. Figure 5b presents the results of the cutback measurements for all 121 cores, where each square in the image represents a core of the MCF. The alignment of the attenuation matrix in this image is the same as the alignment in Fig. 4, where it can be seen that higher loss cores result in lower numbers of counts. Our characterization results indicate that the difference in attenuation between the highest and lowest loss MCF cores is ≈0.04 dB m^−1^. After 290 m of MCF, this difference would result in an ≈12 dB difference in the counts expected from these cores, as is seen in Fig. 4.

### Photonic lantern fabrication

To fabricate the PL, the MCF was threaded into a fluorine-doped silica capillary, which has a lower refractive index compared with the pure silica cladding of the MCF. The capillary was collapsed, by surface tension, on top of the MCF using an oxybutane flame. The cladded structure was then softened, using a similar flame, and stretched by a tapering rig, forming a biconical fibre-like structure. Finally, the multimode port of the PL was revealed by cleaving the centre of the tapered waist. The resultant multicore-to-multimode taper was ≈4 cm long, with an approximately linear profile. The multimode port's core diameter was ≈35 μm and its numerical aperture was 0.22. According to equation (6) in ref. 4, the multimode port therefore supports 585 modes at 500 nm and 406 modes at 600 nm, and so is highly overmoded compared with the 121 single modes of the MCF.

### Photonic lantern insertion loss

The insertion loss of the PL was investigated in two ways. The first technique provided an approximate value for the combined loss of the PL when coupling light from the MCF, to the multimode port, and then back to the MCF. This measurement involved coupling 532 nm light into all of the cores of the MCF at once, and measuring the change in transmission as the PL was made. Using this technique, we measured a total loss of 0.13 dB. The second technique was aimed at investigating the loss experience by light travelling in each of the MCF cores to the multimode output port. To do this, we used a cutback technique (as described above) using 550 nm light to evaluate the loss of 20 m of MCF with the lantern attached at the output end. For each input core, the evaluated loss was then compared with that expected due to the 20 m of MCF alone, using the core-specific propagation losses reported in ‘Methods' section. The difference between the measured and predicted loss is due to the PL. The average loss we inferred for the lantern using this technique was 0.19 dB, and the s.d. of the measurements was ±0.34 dB.

**Figure 6:**
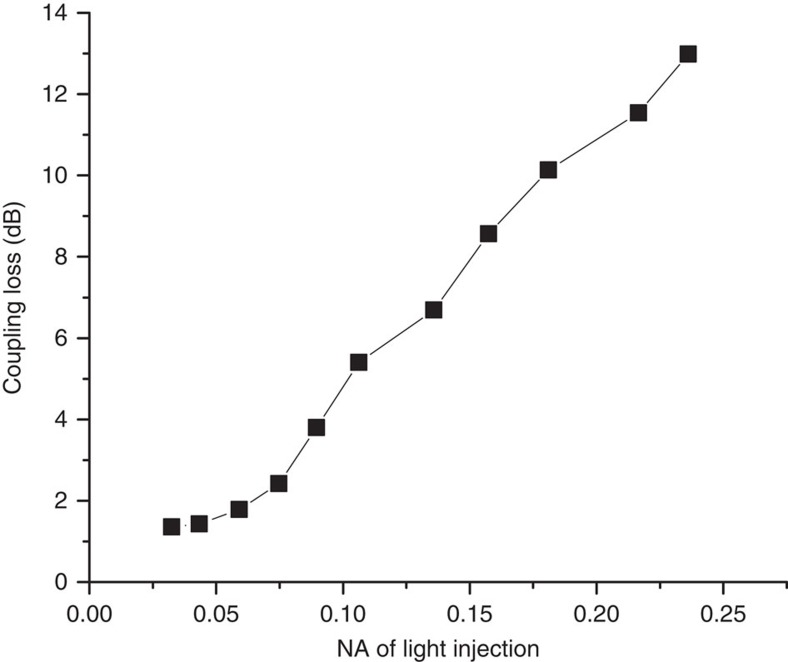
Effect of injection numerical aperture on PL coupling. Coupling loss to the PL as a function of the numerical aperture of the light injection into the multimode port.

The propagation of light when injected into the multimode port of the PL using different numerical apertures was also investigated. Light at 550 nm was collimated, projected through an adjustable iris and focused onto the multimode end of the PL, which was attached to 20 m of MCF. The numerical aperture of the injected light could be adjusted by controlling the size of the iris. After removing the contributions due to the propagation losses of the MCF cores and the Fresnel reflections at the lantern input and MCF output, we obtained the variation of coupling efficiency with injection numerical aperture. The results of these measurements are presented in Fig. 6, where it can be seen that the coupling loss increases with increasing numerical aperture, even though all but the highest value of numerical aperture used are below the 0.22 NA of the multimode port itself. The origin of this increase is that the PL is not mode matched at 550 nm (see ‘Methods' section). Assuming perfect adiabaticity, this means that only the 121 lowest order spatial modes in the multimode port will couple to the single-mode cores of the MCF. As the numerical aperture of the injection is increased, increasingly high-order spatial modes are excited in the multimode port, an increasing fraction of which are not coupled to the MCF cores. As can be seen in Fig. 6, the insertion loss reaches a minimum value of ≈1.3 dB when injecting the light using an numerical aperture of 0.024. This value is still ≈1.0 dB higher than the values measured using the techniques discussed above, and is likely due to imperfect mode matching between the input spot (which is generated by focusing a near-top-hat beam profile), and the spatial modes of the multimode port of the lantern.

### Fill factor enhancement

**Figure 7:**
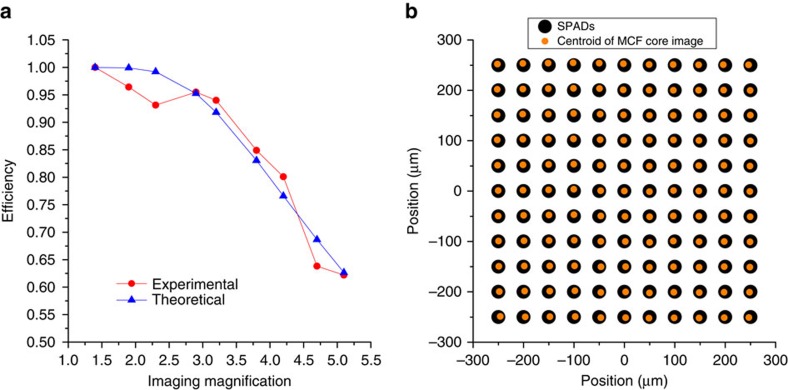
MCF to detector array coupling efficiency. (**a**) Efficiency of core-to-SPAD coupling for a single core as a function of magnification. An efficiency of 1 indicates all of the light from the core falls within the active area of the SPAD. (**b**) A visual representation of how the MCF cores could be imaged to the perfectly square array of SPADs to achieve optimum coupling. The sizes of the symbols do not represent the physical sizes of the MCF modes or the SPADs.

The PL enables the efficient reformatting of multimode light into an array of single-mode cores that can be efficiently coupled to the SPADs on the Megaframe. This effectively increases the fill factor of a subset of the SPADs. To determine the effective fill factor, 532 nm light was injected into the multimode end of a PL attached to 9 m of MCF. Light from one single-mode core at the output of the MCF was imaged onto one SPAD with a magnification *M* ranging from 1.4 to 5.1, and the SPAD counts in each case were recorded. *M* was measured by imaging the MCF cores onto a camera confocal with the SPAD array, and comparing the imaged core spacing to the 10.53 μm spacing reported in ‘Methods' section. We estimate the uncertainty in *M* to be 3%. Since the SPADs are separated by 50 μm, the MCF core spacing should be magnified by *M*=4.75 to simultaneously couple the 121 cores to 121 SPADs, which matches one particular measured *M* value (*M*=4.7) to within experimental error. The SPAD counts were recorded for each *M*, and were normalized by the counts for *M*=1.4 to give the single-core-to-SPAD coupling efficiencies plotted in Fig. 7a. Since each core supports a mode with a 1/*e*^2^ mode field diameter of 1.84 μm, see ‘Methods' section, it is reasonable to assume that all of the light falls inside the ≈6 μm diameter photosensitive area of a SPAD when magnified by just 1.4 (ref. 25). For *M*=4.7, the effective single-core coupling efficiency is 64%, which would be a great improvement over the 1% fill factor without the PL. We were also able to confirm that, for a magnification of *M*=4.7, there was negligible leakage of light from an MCF core to any neighbouring SPAD it was not intended to be coupled with.

To estimate the diameter of the SPAD's active area, we represented the fibre's mode by a circular Gaussian beam of 1.85 μm diameter (see ‘Methods' section), but dilated by *M*=4.75. The fraction of this beam that passes through a concentric circular aperture (representing the SPAD's active area) was calculated as a function of *M* and fitted to the data in Fig. 7a using a *χ*^2^ procedure. The best fit was obtained for an aperture of 6.3 μm diameter, close to the ≈6 μm diameter expected^25^.

Clearly, the single-core 64% coupling efficiency can only be achieved for all cores simultaneously if they lie on a perfect square grid matching the SPAD array. The analysis described above in ‘Methods' section was used to estimate how the overall coupling efficiency of the PL to the SPAD array is degraded by the deviation of the actual MCF's core array from a perfect square grid, represented visually by Fig. 7b. The *M*=4.75 magnification of the fibre image dilates the 0.54 μm RMS displacement of the cores to 2.58 μm. To assess the impact of such a misalignment on the coupling from the MCF to the SPAD array, we again calculated the fraction of a circular Gaussian beam that passes through a circular aperture (of diameter 6.3 μm), but this time with the 121 different mode-SPAD lateral offsets. The simulated average coupling efficiency was 45.8%. (We note this is an underestimate of what could be achieved because, as discussed in ‘Methods' section, the fitting of the MCF modes to the SPAD array is only a partially optimized upper bound.)

### TCSPC experimental details

The TCSPC experimental set-up is shown in Fig. 2a. The TCSPC timer in each pixel of the SPAD array is designed to work in reversed start–stop mode, where the detection of a single-photon at the SPAD array starts the timer and the detection of a trigger pulse stops the measurement. The set-up therefore consists of start and stop arms. The start arm contains the PL and the 290 m long MCF. The optical signal for the start arm is obtained using a femtosecond laser (Fianium HE-1060-1 μJ-fs), operating at a pulse repetition rate of 500 kHz and emission wavelength of 1,064 nm, to generate a broadband supercontinuum by pumping a 20 cm long length of photonic crystal fibre^34^. The supercontinuum pulses are spectrally filtered using bandpass, SP and LP filters (Thorlabs—FEL0500, FES0600 and FESH0700). For calibration of the WTM process, narrow band pulses could be further selected by inserting a high-order 550 nm FP interference filter (developed by Edinburgh Biosciences and Delta Optical Thin Films) into the beam path, together with bandpass filters from Thorlabs (FWHM of 10 nm). The pulses in the start arm were focused onto the multimode end of the PL. At the output of the MCF, an achromatic lens was used to image the 121 MCF single-mode cores onto an 11 × 11 array subset of pixels on the Megaframe SPAD array. A conventional spectrometer (Ocean Optics—USB2000+) was used to record the central wavelength of the pulses passed by the FP interference filter. For the broadband measurements with the diffuser plate, the diffuser plate was rotated at 20 Hz using a chopper motor. The stop arm of the set-up consists of an OCFD (OCF401—Becker & Hickl), which detects the arrival of a pump laser pulse and converts it into a stable electrical trigger signal to stop the TCSPC measurement. To match the time delay of 1.33 μs introduced by the 290 m long fibre in the start arm, a digital delay generator (DG645—Stanford Research Systems) was used to electronically delay the output pulse from the OCFD. Each SPAD used in the experiment detected up to 0.0002 photons per pulse. All measurements were therefore obtained in the photon-starved regime required for TCSPC. Acquiring the spectra took 480 s, due to the low pulse repetition rate of the laser *f*_Laser_=500 kHz.

### Data availability

Raw data can be obtained from the Heriot-Watt PURE data repository system, using the following digital object identifiers. Raw data for calibration of wavelength-to-time-mapping (Fig. 3): 10.17861/cea7ca25-d4f5-4a5e-9e97-f75985378867. Raw data used to evaluate the effective spectral resolution of the wavelength-to-time mapped spectra: 10.17861/de41ea82-2f00-4fdf-b63c-42a1c7c6b607. Raw data for broadband wavelength-to-time-mapping measurements (Fig. 4): 10.17861/9f56de62-81b8-48c1-b373-180f9ba0b52c. Raw data used to evaluate the relative positions of the MCF cores (see ‘Methods' section): 10.17861/5fefc525-7a0b-4f30-aeb1-cdb8a5b2cf9f. Image files used to evaluate wavelength-dependent coupling in the MCF (see ‘Methods' section) 10.17861/4983538a-01b1-45c7-8623-72571a5836f5. Raw data used to evaluate the average mode field diameter for the MCF at 550 nm (Methods): 10.17861/2034cf36-3442-4774-ae65-a9b610aefb91. Raw data used for evaluating the core-dependent MCF propagation loss at 550 nm (Methods): 10.17861/4f6aecc1-6f6f-4f9f-a24e-39001804a8c5. Raw data obtained to evaluate the effect of injection numerical aperture on the coupling efficiency to the multimode port of the PL (see ‘Methods' section): 10.17861/53bdb56c-9045-4ef3-ad4c-c26b01fbda17. Raw data used to evaluate the fill factor enhancement (see ‘Methods' section): 10.17861/5e68f0b7-abf0-470c-a784-b7f42cb7dd34.

## Additional information

**How to cite this article:** Chandrasekharan, H. K. *et al*. Multiplexed single-mode wavelength-to-time mapping of multimode light. *Nat. Commun.* **8**, 14080 doi: 10.1038/ncomms14080 (2017).

**Publisher's note:** Springer Nature remains neutral with regard to jurisdictional claims in published maps and institutional affiliations.
